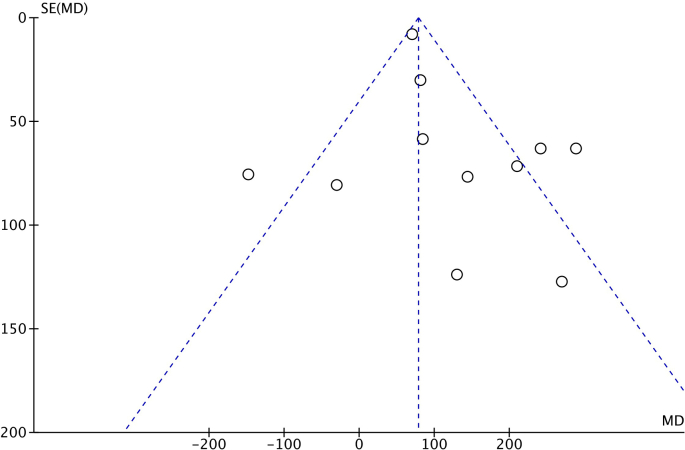# Intravenous *versus* perineural dexamethasone to prolong analgesia after interscalene brachial plexus block: a systematic review with meta-analysis and trial sequential analysis

**DOI:** 10.1016/j.bja.2024.03.042

**Published:** 2024-05-23

**Authors:** Eric Albrecht, Yves Renard, Neel Desai

**Affiliations:** 1 Department of Anaesthesia, Lausanne University Hospital and University of Lausanne, Lausanne, Switzerland; 2 Department of Anaesthesia, Guy's and St Thomas' NHS Foundation Trust, London, UK; 3 King's College London, London, UK

**Keywords:** anaesthetic adjuvants, analgesia, dexamethasone, interscalene nerve block, postoperative pain

## Abstract

**Background:**

The efficacy of perineural *vs* intravenous dexamethasone as a local anaesthetic adjunct to increase duration of analgesia could be particular to specific peripheral nerve blocks because of differences in systemic absorption depending on the injection site. Given this uncertainty, we performed a systematic review with meta-analysis and trial sequential analysis comparing dexamethasone administered perineurally or intravenously combined with local anaesthetic for interscalene brachial plexus block.

**Methods:**

Following a search of various electronic databases, we included 11 trials (1145 patients). The primary outcome was the duration of analgesia defined as the time between peripheral nerve block or onset of sensory blockade and the time to first analgesic request or initial report of pain.

**Results:**

The primary outcome, duration of analgesia, was greater in the perineural dexamethasone group, with a mean difference (95% confidence interval) of 122 (62–183) min, *I*^2^=73%, *P*<0.0001. Trial sequential analysis indicated that firm evidence had been reached. The quality of evidence was downgraded to low, mainly because of moderate inconsistency and serious publication bias. No significant differences were present for any of the secondary outcomes, except for onset time of sensory and motor blockade and resting pain score at 12 h, but the magnitude of differences was not clinically relevant.

**Conclusions:**

There is low-quality evidence that perineural administration of dexamethasone as a local anaesthetic adjunct increases duration of analgesia by an average of 2 h compared with intravenous injection for interscalene brachial plexus block. Given the limited clinical relevance of this difference, the off-label use of perineural administration, and the risk of drug crystallisation, we recommend intravenous dexamethasone administration.

**Systematic review protocol:**

PROSPERO (CRD42023466147).

**Editor's key points**

• Dexamethasone can prolong the duration of analgesia produced by local anaesthetics used for peripheral nerve blocks, but it is unclear whether perineural or intravenous administration is superior.
• The authors performed a systematic review with meta-analysis and trial sequential analysis to compare perineural with intravenous dexamethasone administration combined with local anaesthetic for interscalene nerve block.
• The duration of analgesia was about 2 h longer with perineural *vs* intravenous dexamethasone.
• As perineural use of dexamethasone is off-label and can lead to crystallisation when co-injected with local anaesthetic, the authors recommend intravenous administration given the limited increase in duration of analgesia afforded by perineural administration.

Local anaesthetic adjuncts offer the opportunity to prolong analgesia after administration of local anaesthetics for peripheral nerve blocks.^1^^,^^2^ Among the different adjuvants, dexamethasone possesses the most favourable profile with minimal adverse effects, and has the potential to prolong analgesia for up to 8 h.^3^, ^4^, ^5^ There is conflicting evidence regarding the optimal route of administration of dexamethasone: perineural or intravenous injection.^6^, ^7^, ^8^, ^9^, ^10^ Baeriswyl and colleagues^6^ and Hussain and colleagues^10^ did not find any differences between perineural and intravenous dexamethasone, but the two other systematic reviews reported an increased duration of analgesia of 4 h in favour of the perineural route.^8^^,^^9^ However, these four meta-analyses revealed a high degree of heterogeneity that was not explained by different subgroup analyses, and included various peripheral nerve blocks performed in several anatomical regions, limiting the relevance of the conclusions.

A recent randomised controlled trial that investigated the role of dexamethasone in 182 patients having interscalene brachial plexus block for shoulder surgery determined that intravenous administration was slightly more effective.^11^ As the vascularity of the area blocked could impact systemic absorption, differences in efficacy between perineural and intravascular injection might be block specific and depend on the anatomical location. Given this uncertainty, we performed a systematic review with meta-analysis and trial sequential analysis to elucidate the efficacy of dexamethasone administered perineurally or intravenously when combined with local anaesthetic for interscalene brachial plexus block.

## Methods

This systematic review with meta-analysis adhered to the Preferred Reporting Items for Systematic Reviews and Meta-Analyses (PRISMA) statement,^12^ and was prospectively registered on the International Prospective Register of Systematic Reviews (PROSPERO, CRD42023466147). With the assistance of a medical librarian, we searched the following electronic databases from inception to July 18, 2023: Cochrane Central Register of Controlled Clinical Trials; Ovid Medline; Ovid Embase; and Google Scholar (the latter search limited to the first 300 results). Details of the literature search strategy are described in Supplementary material (Appendix S1). The searches were conducted in accordance with the Peer Review of Electronic Search Strategies (PRESS) checklist and hence included peer review by another medical librarian.^13^ No language or date limits were placed on the search. The references were imported into EndNote™ X9 software (Clarivate™, London, UK) for deduplication. In addition, the authors examined the references of all retrieved citations for any applicable trials that might not have been captured by the above approach. Citations retrieved from the search strategy were entered into a reference management program, Rayyan (Qatar Computing Research Institute, 2016, Doha, Qatar)^14^ and any outstanding duplicate citations were removed. The titles and abstracts of the remaining citations were screened for eligibility by two authors (EA and ND), and the full texts of potentially eligible citations were subsequently assessed for inclusion. Only randomised controlled trials performed on an adult population that compared intravenous to perineural administration of dexamethasone as a local anaesthetic adjunct for interscalene brachial plexus block were included.

For each randomised trial, the methodological quality was evaluated using the Cochrane Collaboration's Risk of Bias tool.^15^ Two authors (EA and YR) used this method to screen, review, and score the items for every trial. Disagreements in extracted data or scoring were adjudicated by a third author (ND). Extracted trial characteristics included: the concentration, nature, and volume of injected local anaesthetic; peripheral nerve block technique; type of surgery; main mode of anaesthesia; postoperative analgesia; and the primary outcome.

The primary outcome was duration of analgesia defined as the time between the peripheral nerve block or onset of sensory blockade and the first analgesic request or initial report of pain. Secondary outcomes were: onset of sensory and motor blockade; duration of motor blockade; pain score at rest and on movement at 6, 12, 24, and 48 h; cumulative i.v. morphine equivalent consumption at 6, 12, 24, and 48 h; incidence of nausea and vomiting at 24 h and in hospital; incidence of postoperative hyperglycaemia, infection and neurological complications; and patient satisfaction. These predefined outcomes were extracted from each trial following the routine approach described for meta-analyses on acute postoperative pain.^16^, ^17^, ^18^ The text, tables or images from the trials were assessed to extract the number of patients, number of events, means, standard error of means, standard deviations, and 95% confidence intervals (95% CI). Data presented graphically were extracted with plot digitising software (Plot Digitizer Version 2.1, Free Software Foundation, Boston, MA, USA). For trials that did not describe sample size or results as means, standard error of the means, standard deviations, or 95% CIs, we contacted the corresponding authors twice by e-mail with a request for access to the complete data set or relevant outcome information. If the corresponding author failed to reply, we used the medians and inter-quartile ranges as approximations of the means and standard deviations, by estimating the means as equivalent to the medians and the standard deviations as the inter-quartile ranges divided by 1.35 or the ranges divided by 4, as recommended.^19^^,^^20^ Opioids were converted to equianalgesic intravenous morphine doses (i.v. morphine 10 mg=oral morphine 30 mg=oral codeine 165 mg=i.v. fentanyl 100 μg=oral hydrocodone 30 mg=i.v. nalbuphine 10 mg=i.v. pethidine 75 mg=i.v. tramadol 100 mg).^21^ For pain or satisfaction scores using an 11-unit numeric, verbal or visual analogue rating scale, results were transposed to a 0–10 analogue scale to permit statistical analysis. The Grades of Recommendation, Assessment, Development and Evaluation (GRADE) system was applied to every outcome to evaluate the quality of evidence.^22^

All meta-analyses were conducted using RevMan 5.4.0 (The Nordic Cochrane Centre, The Cochrane Collaboration 2020, Copenhagen, Denmark). This software estimates the weighted mean differences for continuous data and the risk ratios for categorical data between groups, with an overall estimate of the pooled effect. We performed meta-analysis only if two or more trials reported any given outcome, and calculated the *I*^2^ coefficient to assess heterogeneity and set predetermined limits for low (<50%), moderate (50–74%), and high (>75%) levels.^15^ A random-effects model was applied in circumstances when moderate or high heterogeneity was observed, and we used a fixed-effects model where low heterogeneity was seen.^23^ The results are presented as mean difference or risk ratio with 95% CI, and a two-sided *P*-value <0.05 was set to be significant. To account for sources of heterogeneity, subgroup analyses were conducted for our primary outcomes according to the type of local anaesthetic (bupivacaine, levobupivacaine or ropivacaine) and the reported use of multimodal analgesic treatment inclusive of two different analgesic modalities (yes or no). The risk of publication bias associated with our primary outcome was estimated by drawing a funnel plot of the standard error of the mean difference of duration of analgesia (y-axis) as a function of the mean difference of duration of analgesia (x-axis),^24^ and confirmed with Duval and Tweedie's trim and fill test.^25^ This assessment was undertaken using Comprehensive Meta-analysis version 2 (Biostat, Englewood, NJ, USA). Finally, trial sequential analysis was performed for the primary outcome to confirm whether firm evidence was reached or not (TSA software version 0.9.5.10 Beta; Copenhagen Trial Unit, Center for Clinical Intervention Research, Rigshospitalet, Copenhagen, Denmark).

## Results

**Fig 1:**
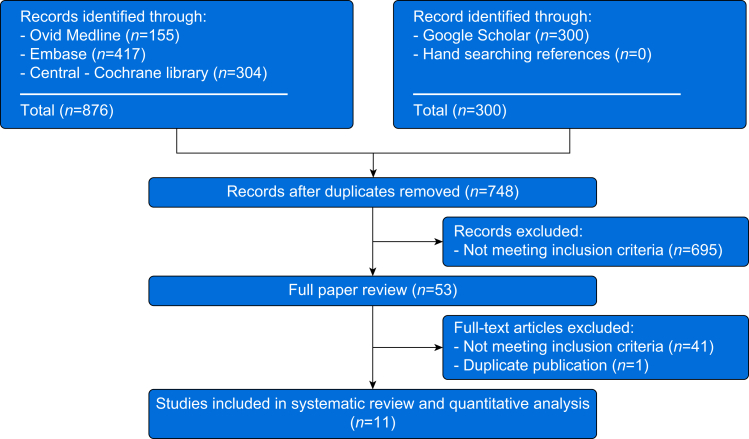
PRISMA flow diagram summarising the retrieved, included, and excluded randomised controlled trials. PRISMA, Preferred Reporting Items for Systematic Reviews and Meta-Analyses.

**Fig 2:**
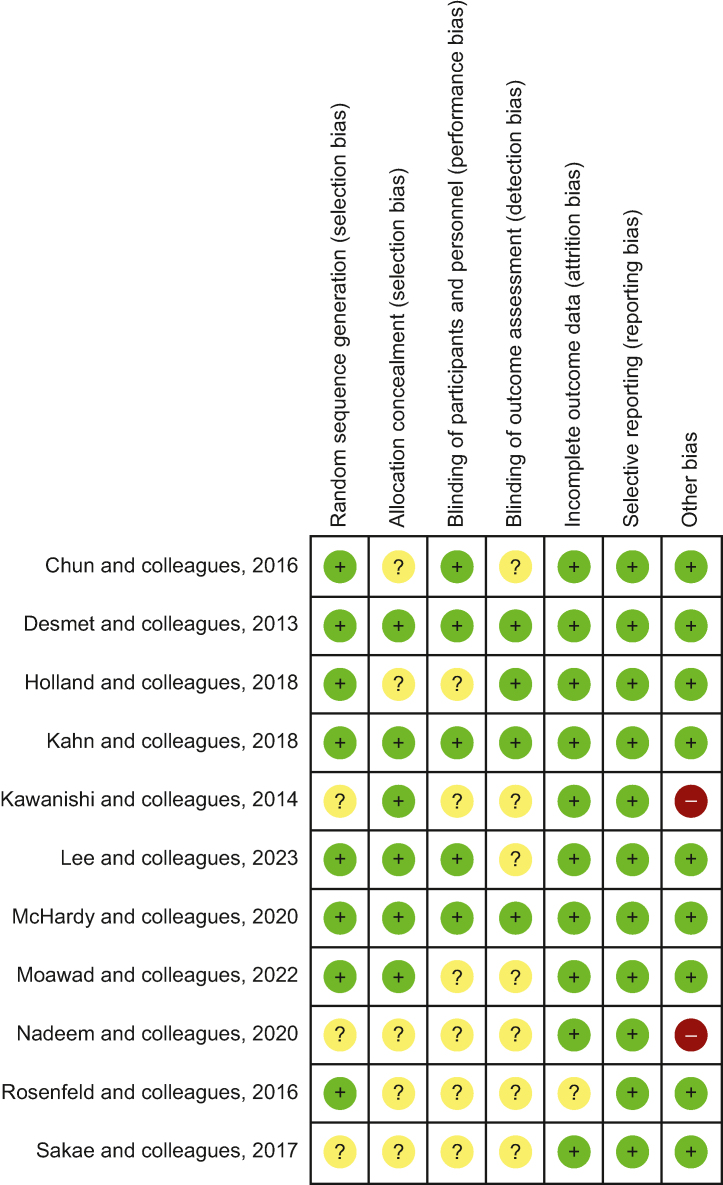
Cochrane Collaboration summary of the risk of bias relevant to each trial. Green circle, low risk of bias; red circle, high risk of bias; yellow circle, unclear risk of bias.

We identified 748 trials and 11 of these,^11^^,^^26^, ^27^, ^28^, ^29^, ^30^, ^31^, ^32^, ^33^, ^34^, ^35^ including a total of 1145 patients, met the inclusion criteria (Fig. 1). The risk of bias of the different trials is summarised in Figure 2. Four corresponding authors were contacted,^27^^,^^28^^,^^31^^,^^32^ and one provided additional data.^27^

Table 1 details the trial characteristics. In eight trials, the authors included patients having shoulder arthroscopy,^11^^,^^26^, ^27^, ^28^, ^29^, ^30^, ^31^^,^^35^ in two, open shoulder and upper arm surgery,^32^^,^^33^ and in one, both.^34^ Patients received ropivacaine in seven trials,^11^^,^^26^^,^^27^^,^^30^^,^^31^^,^^34^^,^^35^ bupivacaine in three,^28^^,^^29^^,^^33^ and levobupivacaine in one.^32^ Interscalene brachial plexus blocks were conducted under ultrasound guidance in all trials except for one.^27^ Surgery was performed under general anaesthesia in eight trials^11^^,^^26^, ^27^, ^28^^,^^30^^,^^31^^,^^34^^,^^35^ and under sedation in one.^29^ The remaining two trials did not specify the main mode of anaesthesia.^32^^,^^33^ Only authors from four trials prescribed postoperative multimodal analgesic treatment.^11^^,^^27^^,^^29^^,^^31^

**Table 1:** Characteristics of the included trials comparing perineural with intravenous dexamethasone. NSAID, non-steroidal anti-inflammatory drug.

Reference	Group (*n*)	Local anaesthetic	Regional technique	Surgery	Main mode of anaesthesia	Postoperative analgesia	Primary outcome
Chun and colleagues, 2016^26^	Perineural 5 mg (50) Intravenous 5 mg (49)	Ropivacaine 0.75%, 8 ml	Ultrasound	Arthroscopic shoulder surgery	General anaesthesia	NSAID and opioid	Time to first analgesic request
Desmet and colleagues, 2013^27^	Perineural 10 mg (49) Intravenous 10 mg (49)	Ropivacaine 0.5%, 30 ml	Nerve stimulation	Arthroscopic shoulder surgery	General anaesthesia	Paracetamol, NSAID, and opioid	Duration of analgesia
Holland and colleagues, 2018^28^	Perineural 4 mg (69) Intravenous 4 mg (70), Perineural 8 mg (70) Intravenous 8 mg (70)	Bupivacaine 0.5%, 30 ml	Ultrasound	Arthroscopic shoulder surgery	General anaesthesia	Not specified	Duration of analgesia
Kahn and colleagues, 2018^29^	Perineural 1 mg (63) Intravenous 1 mg (62)	Bupivacaine 0.5%, 15 ml	Ultrasound	Arthroscopic shoulder surgery	Sedation	Paracetamol, NSAID, and opioid	Time to first pain
Kawanishi and colleagues, 2014^30^	Perineural 4 mg (12) Intravenous 4 mg (10)	Ropivacaine 0.75%, 20 ml	Ultrasound	Arthroscopic shoulder surgery	General anaesthesia	NSAID	Time to first analgesic request
Lee and colleagues, 2023^31^	Perineural 5 mg (36) Intravenous 5 mg (35)	Ropivacaine 0.5%, 12 ml	Ultrasound	Arthroscopic shoulder surgery	General anaesthesia	Paracetamol, NSAID, and opioid	Time to first pain
McHardy and colleagues, 2020^11^	Perineural 4 mg (92) Intravenous 4 mg (90)	Ropivacaine 0.5%, 5 ml	Ultrasound	Arthroscopic shoulder surgery	General anaesthesia	Paracetamol, NSAID, and opioid	Time to first pain
Moawad and colleagues, 2022^32^	Perineural 4 mg (30) Intravenous 4 mg (30)	Levobupivacaine 0.5%, 20 ml	Ultrasound	Shoulder and upper arm surgery	Not specified	Paracetamol and opioid	Duration of sensory block
Nadeem and colleagues, 2020^33^	Perineural 0.15 mg kg^−1^ (45) Intravenous 0.25 mg kg^−1^ (45)	Bupivacaine 2 mg kg^−1^ (concentration not specified)	Ultrasound	Shoulder and upper arm surgery	Not specified	Not specified	Time to first analgesic request
Rosenfeld and colleagues, 2016^34^	Perineural 8 mg (42) Intravenous 8 mg (37)	Ropivacaine 0.5%, 28 ml	Ultrasound	Arthroscopic and open shoulder surgery	General anaesthesia	NSAID and opioid	Duration of sensory block
Sakae and colleagues, 2017^35^	Perineural 4 mg (20) Intravenous 4 mg (20)	Ropivacaine 0.75, 20 ml	Nerve stimulation and ultrasound	Arthroscopic shoulder surgery	General anaesthesia	NSAID and opioid	Duration of sensory block

**Fig 3:**
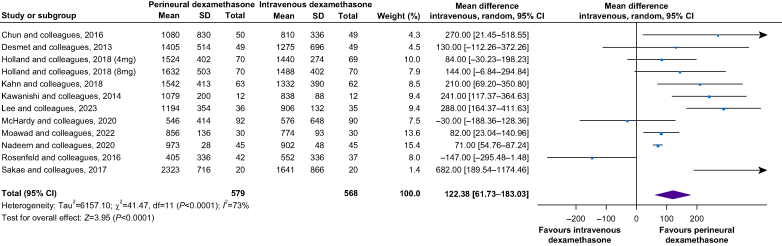
Forest plot of the duration of analgesia. For every trial, the square depicts the risk ratio and the horizontal lines on either side of it represent the 95% CI. The summary result is presented as a diamond. CI, confidence interval; df, degrees of freedom; sd, standard deviation.

**Figure S1:**
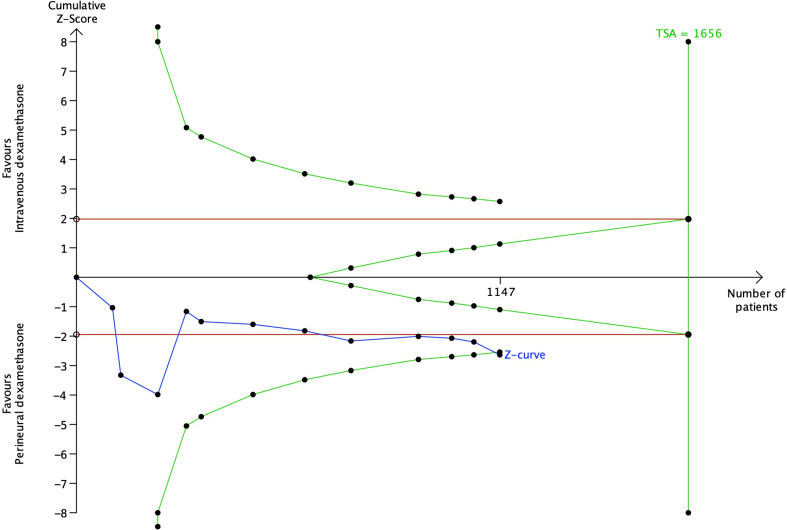


Our primary outcome, duration of analgesia, was increased in the perineural group, with a mean difference (95% CI) of 122 (62–183) min, *I*^2^ = 73%, *P*<0.0001 (Fig. 3), without subgroup differences between bupivacaine, levobupivacaine or ropivacaine (*P*=0.33) and regardless of whether multimodal analgesia was prescribed or not (*P*=0.50). Trial sequential analysis indicated that firm evidence had been reached with regard to the increased duration of analgesia shown with perineural dexamethasone (see also Supplementary Fig. S1). With respect to the risk of publication bias, Duval and Tweedie's trim and fill test indicated a combined studies point estimate (95% CI) of 0.55 (0.24–0.87) with a random-effects model. Using trim and fill, these values were 0.23 (−0.12 to 0.58) and suggested that four trials were missing.

With the exception of pain scores on movement at 6, 12, 24, and 48 h that were not investigated by any of the included trials, all secondary pain-related outcomes are presented in Table 2. The quality of evidence for the primary and secondary outcomes was downgraded to low to very low because of the variable presence of serious limitations, small sample size, moderate inconsistency, and serious publication bias (Table 3).

**Table 2:** Meta-analysis of the secondary outcomes. Values are mean difference, or risk ratio. CI, confidence interval.

Outcome	References	Number of trials	Number of patients		Effect size (95% CI)	*I*^2^ (%)	*P*-value of overall effect
			Perineural	Intravenous			
Duration of motor blockade (h)	McHardy and colleagues, 2020,^11^ Moawad and colleagues 2022,^32^ Sakae and colleagues 2017^35^	3	142	140	120 (−38 to 277)	52	0.14
Onset of sensory blockade (min)	Moawad and colleagues 2022,^32^ Nadeem and colleagues 2020,^33^ Sakae and colleagues 2017^35^	3	95	95	−1 −1 to −1)	30	<0.0001
Onset of motor blockade (min)	Moawad and colleagues 2022,^32^ Nadeem and colleagues 2020^33^	2	75	75	−1 (−2 to −1)	57	0.002
Pain score at rest at 6 h (0–10)	Chun and colleagues 2016,^26^ Moawad and colleagues 2022^32^	2	80	79	0.0 (−0.3 to 0.3)	0	1
Pain score at rest at 12 h (0–10)	Chun and colleagues 2016,^26^ Lee and colleagues 2023,^31^ McHardy and colleagues 2020,^11^ Moawad and colleagues 2022,^32^ Sakae and colleagues 2017^35^	5	228	224	−0.7 (−1.0 to −0.4)	40	<0.0001
Pain score at rest at 24 h (0–10)	Kawanishi and colleagues 2014,^30^ Lee and colleagues 2023,^31^ McHardy and colleagues 2020,^11^ Moawad and colleagues 2022,^32^ Sakae and colleagues 2017^35^	5	220	216	−0.3 (−0.9 to 0.3)	49	0.3
Pain score at rest at 48 h (0–10)	Chun and colleagues 2016,^26^ Kahn and colleagues 2018,^29^ Lee and colleagues 2023^31^	3	149	144	0.1 (−0.2 to 0.4)	0	0.45
Cumulative intravenous morphine equivalent consumption at 24 h (mg)	Holland and colleagues 2018,^28^ Lee and colleagues 2023,^31^ McHardy and colleagues 2020,^11^ Moawad and colleagues 2022,^32^ Rosenfeld and colleagues 2016^34^	6	309	241	0 (−1 to 0)	37	0.14
Cumulative intravenous morphine equivalent consumption at 48 h (mg)	Kahn and colleagues 2018,^29^ Lee and colleagues 2023,^31^	2	99	96	3 (−3 to 9)	50	0.28
Rate of in hospital postoperative nausea and vomiting (%)	Kawanishi and colleagues 2014,^30^ Lee and colleagues 2023,^31^ Moawad and colleagues 2022,^32^ Rosenfeld and colleagues 2016,^34^ Sakae and colleagues 2017^35^	5	202	194	0.93 (0.66–1.32)	0	0.7
Patient satisfaction (0–10)	Kahn and colleagues 2018,^29^ Rosenfeld and colleagues 2016^34^	2	101	95	−0.4 (−1.0 to 0.2)	0	0.15

**Table 3:** GRADE quality of evidence assessment for each outcome. GRADE, Grades of Recommendation, Assessment, Development and Evaluation. ∗Of note the trial sequential analysis revealed that enough patients were included to establish evidence. ^†^*I*^2^ was above 50% with wide variance of point estimates across studies. Final decision to rate down quality of evidence by one level for moderate inconsistency. ^‡^Final decision to rate down quality of evidence by one level for serious publication bias. ^¶^Final decision to rate down quality of evidence by one level for serious limitation. ^§^Wide confidence interval with potential clinical impact. Final decision to rate down quality of evidence by one level for serious imprecision.

Outcome	Limitations	Inconsistency	Indirectness	Imprecision	Publication bias	Total number of participants	Conclusion	Quality of evidence
Duration of analgesia (h)	No serious limitations∗	Moderate inconsistency^†^	No serious indirectness	No serious imprecision	Serious publication bias^‡^	1147	Perineural superior to intravenous dexamethasone	Low quality (⊕⊕OO)
Duration of motor blockade (h)	Small sample size^¶^	Moderate inconsistency^†^	No serious indirectness	Serious imprecision^§^	Serious publication bias^‡^	282	No difference between perineural and intravenous dexamethasone	Very low quality (⊕OOO)
Onset of sensory blockade (min)	Small sample size^¶^	No inconsistency	No serious indirectness	No serious imprecision	Serious publication bias^‡^	190	Perineural superior to intravenous dexamethasone	Low quality (⊕⊕OO)
Onset of motor blockade (min)	Small sample size^¶^	Moderate inconsistency^†^	No serious indirectness	No serious imprecision	Serious publication bias^‡^	150	Perineural superior to intravenous dexamethasone	Very low quality (⊕OOO)
Pain score at rest at 6 h (0–10)	Small sample size^¶^	Not applicable	No serious indirectness	No serious imprecision	Serious publication bias^‡^	159	No difference between groups	Low quality (⊕⊕OO)
Pain score at rest at 12 h (0–10)	Small sample size^¶^	No inconsistency	No serious indirectness	No serious imprecision	Serious publication bias^‡^	452	Perineural superior to intravenous dexamethasone	Low quality (⊕⊕OO)
Pain score at rest at 24 h (0–10)	Small sample size^¶^	No inconsistency	No serious indirectness	No serious imprecision	Serious publication bias^‡^	436	No difference between perineural and intravenous dexamethasone	Low quality (⊕⊕OO)
Pain score at rest at 48 h (0–10)	Small sample size^¶^	No inconsistency	No serious indirectness	No serious imprecision	Serious publication bias^‡^	293	No difference between perineural and intravenous dexamethasone	Low quality (⊕⊕OO)
Cumulative intravenous morphine equivalent consumption at 24 h (mg)	Small sample size^¶^	No inconsistency	No serious indirectness	No serious imprecision	Serious publication bias^‡^	550	No difference between perineural and intravenous dexamethasone	Low quality (⊕⊕OO)
Cumulative intravenous morphine equivalent consumption at 48 h (mg)	Small sample size^¶^	Moderate inconsistency^†^	No serious indirectness	No serious imprecision	Serious publication bias^‡^	195	No difference between perineural and intravenous dexamethasone	Very low quality (⊕OOO)
Rate of in hospital postoperative nausea and vomiting (%)	Small sample size^¶^	No inconsistency	No serious indirectness	No serious imprecision	Serious publication bias^‡^	396	No difference between perineural and intravenous dexamethasone	Low quality (⊕⊕OO)
Patient satisfaction (0–10)	Small sample size^¶^	No inconsistency	No serious indirectness	No serious imprecision	Serious publication bias^‡^	196	No difference between perineural and intravenous dexamethasone	Low quality (⊕⊕OO)

In the four trials that measured glycaemia,^11^^,^^26^^,^^27^^,^^33^ two reported an increased value in the intravenous compared with the perineural group with a mean difference of 0.3–0.4 mM^−1^.^11^^,^^33^ Desmet and colleagues^27^ reported no infections or neurological deficits, whereas Holland and colleagues^28^ reported that 0.02% of patients had persistent paraesthesia at 6 months subsequent to surgery without differences between groups.

## Discussion

This systematic review and meta-analysis with trial sequential analysis compared dexamethasone administered perineurally or intravenously as a local anaesthetic adjunct for interscalene brachial plexus block. Based on 11 trials and 1145 patients, we concluded that there was a low level of evidence that the perineural route increases duration of analgesia by a mean of 2 h compared with the intravenous route, without subgroup differences between bupivacaine, levobupivacaine or ropivacaine and regardless of whether multimodal analgesia was prescribed or not. Further, there was also low to very low level of evidence that perineural dexamethasone decreased the onset time of sensory and motor blockade and rest pain score at 12 h, but the magnitude of difference was not of clinical relevance. Importantly, we were able to reduce the degree of heterogeneity by focusing on a specific block with subgroup analyses.

Perineural injection of dexamethasone is an off-label route of administration. Many studies, however, have demonstrated reassuring findings and found no neurological complications associated with the use of perineural dexamethasone.^36^, ^37^, ^38^ Even if dexamethasone were to be safe, any preservative present in the vial could be neurotoxic and hence a preservative-free solution should be used.^39^ Dexamethasone, however, is not compatible with ropivacaine. Indeed, Watkins and colleagues^40^ reported the occurrence of crystallisation when these drugs were mixed together in the same syringe because of the incompatibility of ropivacaine with alkaline solutions and the elevated pH of dexamethasone.^40^ Given this, to administer dexamethasone perineurally with ropivacaine, it is safest, in the opinion of the authors, to proceed with sequential injection even if crystallisation might occur *in situ*. Taking into account the disputable clinical relevance of a 2-h difference in the duration of analgesia, the off-label route of perineural administration, and the risk of crystallisation, we recommend that dexamethasone be administered intravenously.

Hypotheses for the mechanism of action of perineural dexamethasone in prolonging the duration of analgesia include: decreased activity of the nociceptive C-fibres^41^; inhibition of neuronal potassium channels^42^; reduced absorption of the local anaesthetic secondary to a local vasoconstrictive effect^43^; or a systemic anti-inflammatory effect after vascular uptake.^44^ A recent randomised controlled trial in volunteers that investigated the mechanism of action of dexamethasone suggested a local rather than systemic effect.^45^ The translation of these results into clinical practice, however, is limited owing to the absence of inflammation subsequent to the lack of surgical insult in the healthy participants.

This meta-analysis and systematic review has some limitations. Firstly, many of the secondary pain-related outcomes that we planned to examine were not investigated by the included trials. Secondly, some outcomes were only reported by a few trials. Moreover, we focused solely on the interscalene brachial plexus block, and therefore our conclusions should not be generalised to peripheral nerve blocks in different anatomical locations with variable absorption characteristics secondary to the potentially distinctive vascularity of the area blocked. Finally, we did not perform a subgroup analysis related to the volume of local anaesthetic administered, which might have partly explained the heterogeneity.

In conclusion, we found low-quality evidence that perineural administration of dexamethasone as a local anaesthetic adjunct increases the duration of analgesia by an average of 2 h compared with intravenous injection for interscalene brachial plexus block. In view of the limited clinical relevance of this result, the off-label route of perineural administration, and the risk of crystallisation, we recommend administration of dexamethasone intravenously. Should dexamethasone be injected with ropivacaine via the perineural route, we recommend sequential injection to minimise the impact of crystallisation.

## Authors’ contributions

Study design: EA, ND

Data interpretation: EA, ND

Manuscript preparation: EA

Data collection, statistical analyses: YR

Manuscript editing: YR, ND

## Acknowledgements

We are grateful to C. Jaques (Medical Library, Research and Education Department, Lausanne University Hospital, Switzerland) for her assistance in the literature search.

## Declarations of interest

EA has received grants from the Swiss Academy for Anaesthesia Research (SACAR), Lausanne, Switzerland, B. Braun Medical AG, Sempach, Switzerland, and the Swiss National Science Foundation to support his clinical research. EA has further received honoraria from B. Braun Medical AG Switzerland, Sintetica Ltd UK, and MSD AG Switzerland. The other authors declare that they have no conflicts of interest.

## Funding

Departmental funding (Department of Anaesthesia, University Hospital of Lausanne, Lausanne, Switzerland).

## Figures and Tables

**Figure S2:**