# Supplementary material for: Intravenous versus perineural dexamethasone to prolong analgesia after interscalene brachial plexus block: a systematic review with meta-analysis and trial sequential analysis
Source: Br J Anaesth. 2024 May 23;133(1):135–45. doi: 10.1016/j.bja.2024.03.042 (PMC11213995; doi:10.1016/j.bja.2024.03.042)
Supplement: Multimedia component 2 [file mmc2.docx]

**Supporting Information**

Additional supporting information can be found online in supplemenatary materials.

**Appendix S1.** Details of the literature search strategy.

**Figure S1.** Trial sequential analysis for the duration of analgesia. The cumulative Z curve (blue) crosses the trial sequential boundary curve (green), indicating that firm evidence has been reached to conclude that the perineural administration of dexamethasone is superior to the intravenous route of administration.

**Figure S2.** Funnel plot of the duration of analgesia.
